# Sample Size Reassessment and Hypothesis Testing in Adaptive Survival Trials

**DOI:** 10.1371/journal.pone.0146465

**Published:** 2016-02-10

**Authors:** Dominic Magirr, Thomas Jaki, Franz Koenig, Martin Posch

**Affiliations:** 1 Section of Medical Statistics, Center for Medical Statistics, Informatics, and Intelligent Systems, Medical University of Vienna, Vienna, Austria; 2 Medical and Pharmaceutical Statistics Research Unit, Lancaster University, Lancaster, United Kingdom; Cardiff University, UNITED KINGDOM

## Abstract

Mid-study design modifications are becoming increasingly accepted in confirmatory clinical trials, so long as appropriate methods are applied such that error rates are controlled. It is therefore unfortunate that the important case of time-to-event endpoints is not easily handled by the standard theory. We analyze current methods that allow design modifications to be based on the full interim data, i.e., not only the observed event times but also secondary endpoint and safety data from patients who are yet to have an event. We show that the final test statistic may ignore a substantial subset of the observed event times. An alternative test incorporating all event times is found, where a conservative assumption must be made in order to guarantee type I error control. We examine the power of this approach using the example of a clinical trial comparing two cancer therapies.

## 1 Introduction

There are often strong ethical and economic arguments for conducting interim analyses [1] of an ongoing clinical trial and for making changes to the design if warranted by the accumulating data. One may decide, for example, to increase the sample size on the basis of promising interim results. Or perhaps one might wish to drop a treatment from a multi-arm study on the basis of unsatisfactory safety data. Owing to the complexity of clinical drug development, it is not always possible to anticipate the need for such modifications, and therefore not all contingencies can be dealt with in the statistical design.

Unforeseen interim modifications complicate the frequentist statistical analysis of the trial considerably. Over recent decades many authors have investigated so-called “adaptive designs” in an effort to maintain the concept of type I error control [2–6]. While the theory behind these methods is now well understood if responses are observed immediately, subtle problems arise when responses are delayed, e.g., in survival trials.

[7] proposed adaptive survival tests that are constructed using the independent increments property of logrank test statistics [8–10]. However, as pointed out by [11], these methods only work if interim decision making is based solely on the interim logrank test statistics and any secondary endpoint data from patients who have already had an event. In other words, investigators must remain blind to the data from patients who are censored at the interim analysis. [12] argue that decisions regarding interim design modifications should be as substantiated as possible, and propose a test procedure that allows investigators to use the full interim data. This methodology, similar to that of [13], does not require any assumptions regarding the joint distribution of survival times and short-term secondary endpoints, as do, e.g., the methods proposed by [14], [15, 16] and [17].

In this article we analyze the proposals of [13] and [12] and show that they are both based on weighted inverse-normal test statistics [18], with the common disadvantage that the final test statistic may ignore a substantial subset of the observed survival times. This is a serious limitation, as disregarding part of the observed data is generally considered inappropriate even if statistical error probabilities are controlled—see, for example, the discussion on overrunning in group sequential trials [17]. We quantify the potential inflation of the type I error rate if all observed data were used in these approaches. By adjusting the critical boundaries for the least favourable scenario we derive an alternative testing procedure which allows both, sample size reassessment and the use of all observed data.

The article is organized as follows. In Section 2 we review standard adaptive design theory and the recent methods of [13] and [12], as well as calculating the maximum type I error rate if the ignored data is naively reincorporated into the test statistic. In addition we construct a full-data guaranteed level-*α* test. In Section 3 we illustrate the procedures in clinical trial example and discuss the efficiency of the considered testing procedures. We present our conclusions in Section 4. R code to reproduce our results in provided in a supplementary file (S1 File).

## 2 Methods

### 2.1 Adaptive Designs

Comprehensive accounts of adaptive design methodology can be found in [6, 19]. For testing a null hypothesis, *H*_0_: *θ* = 0, against the one-sided alternative, *H*_*a*_: *θ* > 0, the two-stage adaptive test statistic is of the form *f*_1_(*p*_1_) + *f*_2_(*p*_2_), where *p*_1_ is the p-value based on first-stage data, *p*_2_ is the p-value based on second-stage data, and *f*_1_ and *f*_2_ are prespecified monotonically decreasing functions. Consider the simplest case that no early rejection of the null hypothesis is possible at the end of the first stage. We will restrict attention to the weighted inverse-normal test statistic [18],

$$Z=w_{1}\Phi^{-1}\left(1-p_{1}\right)+w_{2}\Phi^{-1}\left(1-p_{2}\right),$$ (1)

where Φ denotes the standard normal distribution function and *w*_1_ and *w*_2_ are prespecified weights such that $w_{1}^{2}+w_{2}^{2}=1$. If *Z* > Φ^−1^(1 − *α*), then *H*_0_ may be rejected at level *α*. The assumptions required to make this a valid level-*α* test are as follows [20].

**Assumption 1**

Let $X_{1}^{\text{int}}$ denote the data available at the interim analysis, where $X_{1}^{\text{int}}\in R^{n}$ with distribution function $G(x_{1}^{\text{int}};\theta )$. In general, $X_{1}^{\text{int}}$ will contain information not only concerning the primary endpoint, but also measurements on secondary endpoints and safety data. It is assumed that the first-stage p-value function *p*_1_: ℝ^*n*^ → [0, 1] satisfies

$$\int_{R^{n}}1\left\{p_{1}\left(x_{1}^{\text{int}}\right)\leq u\right\}\;d\;G\left(x_{1}^{\text{int}};0\right)\leq u\;\text{for}\;\text{all}\;u\in \left[0,1\right].$$

**Assumption 2**

At the interim analysis, a second-stage design *d* is chosen. The second-stage design is allowed to depend on the unblinded first-stage data without prespecifying an adaptation rule. Denote the second-stage data by *Y*, where $Y\in R^{m}$. It is assumed that the distribution function of *Y*, denoted by $F_{\delta ,x_{1}^{\text{int}}}\left(y,\theta \right)$, is known for all possible second stage designs, *δ*, and all first-stage outcomes, $x_{1}^{\text{int}}$.

**Assumption 3**

The second-stage p-value function *p*_2_: ℝ^*m*^ → [0, 1] satisfies $\int_{R^{m}}1\{p_{2}\left(y\right)\leq u\}\;d\;F_{\delta ,x_{1}^{\text{int}}}\left(y;0\right)\leq u\;\text{for}\;\text{all}\;u\in [0,1]$.

#### Immediate responses

The aforementioned assumptions are easy to justify when primary endpoint responses are observed more-or-less immediately. In this case $X_{1}^{\text{int}}$ contains the responses of all patients recruited prior to the interim analysis. A second-stage design *δ* can subsequently be chosen with the responses from a new cohort of patients contributing to *Y*.

#### Delayed responses and the independent increments assumption

**Fig 1:**
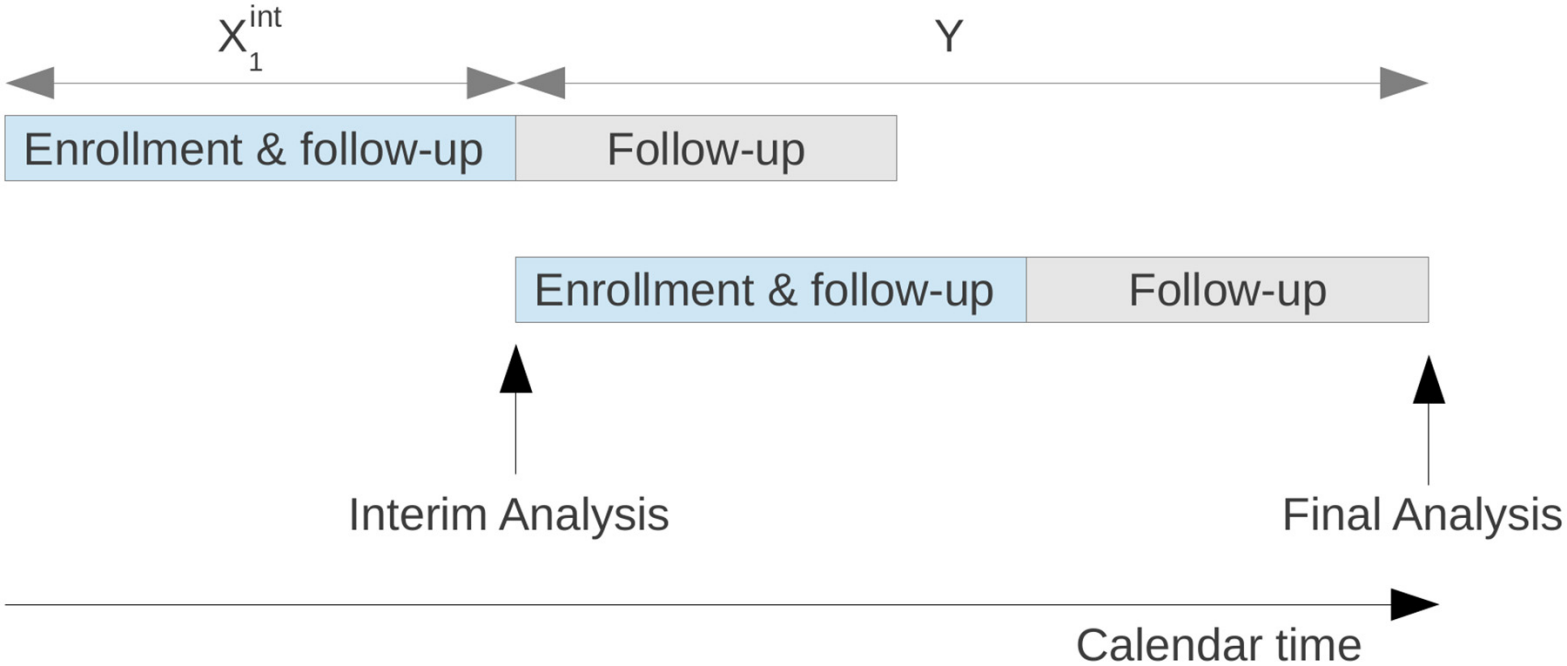
Schematic of a two-stage adaptive trial design with delayed response using the independent increments assumption.

An interim analysis may take place whilst some patients have entered the study but have yet to provide a data point on the primary outcome measure. Most approaches to this problem [7, 8, 10] attempt to take advantage of the well known independent increments structure of score statistics in group sequential designs [21]. As pictured in Fig 1, $X_{1}^{\text{int}}$ will generally include responses on short-term secondary endpoints and safety data from patients who are yet to provide a primary outcome measure, while *Y* consists of some delayed responses from patients recruited prior to the interim analysis, mixed together with responses from a new cohort of patients.

Let $S(X_{1}^{\text{int}})$ and $I(X_{1}^{\text{int}})$ denote the score statistic and Fisher’s information for *θ*, calculated from primary endpoint responses in $X_{1}^{\text{int}}$. Assuming suitable regularity conditions, the asymptotic null distribution of $S(X_{1}^{\text{int}})$ is Gaussian with mean zero and variance $I(X_{1}^{\text{int}})$[22]. The independent increments assumption is that for all first-stage outcomes $x_{1}^{\text{int}}$ and second-stage designs *δ*, the null distribution of *Y* is such that

$$S\left(x_{1}^{\text{int}},Y\right)-S\left(x_{1}^{\text{int}}\right)∼N\left\{0,I\left(x_{1}^{\text{int}},Y\right)-I\left(x_{1}^{\text{int}}\right)\right\},$$ (2)

at least approximately, where $S(X_{1}^{\text{int}},Y)$ and $I(X_{1}^{\text{int}},Y)$ denote the score statistic and Fisher’s information for *θ*, calculated from primary endpoint responses in $\left(X_{1}^{\text{int}},Y\right)$.

Unfortunately, Eq (2) is seldom realistic in an adaptive setting. [11] show that if the adaptive strategy at the interim analysis is dependent on short-term outcomes in $X_{1}^{\text{int}}$ that are correlated with primary endpoint outcomes in *Y*, i.e., from the same patient, then a naive appeal to the independent increments assumption can lead to very large type I error inflation.

#### Delayed responses with patient-wise separation

An alternative approach, which we call “patient-wise separation”, redefines the first-stage p-value, *p*_1_: ℝ^*p*^ → [0, 1], to be a function of *X*_1_, where *X*_1_ denotes all the data from patients recruited prior to the interim analysis at calendar time *T*^int^, followed-up until a pre-fixed maximum calendar time *T*^max^. In this case *p*_1_ may not be observable at the time the second-stage design *δ* is chosen. This is not a problem, as long as no early rejection at the end of the first stage is foreseen. Any interim decisions, such as increasing the sample size, do not require knowledge of *p*_1_. It is assumed that *Y* consists of responses from a new cohort of patients, such that $x_{1}^{\text{int}}$ could be formally replaced with *x*_1_ in the aforementioned adaptive design assumptions. We call this patient-wise separation because data from the same patient cannot contribute to both *p*_1_ and *p*_2_.

[23] and [24] apply this approach when a patient’s primary outcome can be measured after a fixed period of follow-up, e.g., 4 months. However, one must take additional care with a time-to-event endpoint, as one is typically not prepared to wait for all first-stage patients—those patients recruited prior to *T*^int^—to have an event. Rather, *p*_1_ is defined as the p-value from a statistical test applied to the data from first-stage patients followed up until time *T*^end^, for some *T*^end^ ≤ *T*^max^. In this case it is vital that *T*^end^ be fixed at the start of the trial, either explicitly or implicitly [12, 13]. Otherwise, if *T*^end^ were to depend on the adaptive strategy at the interim analysis, this would impact the distribution of *p*_1_ and could lead to type I error inflation.

**Fig 2:**
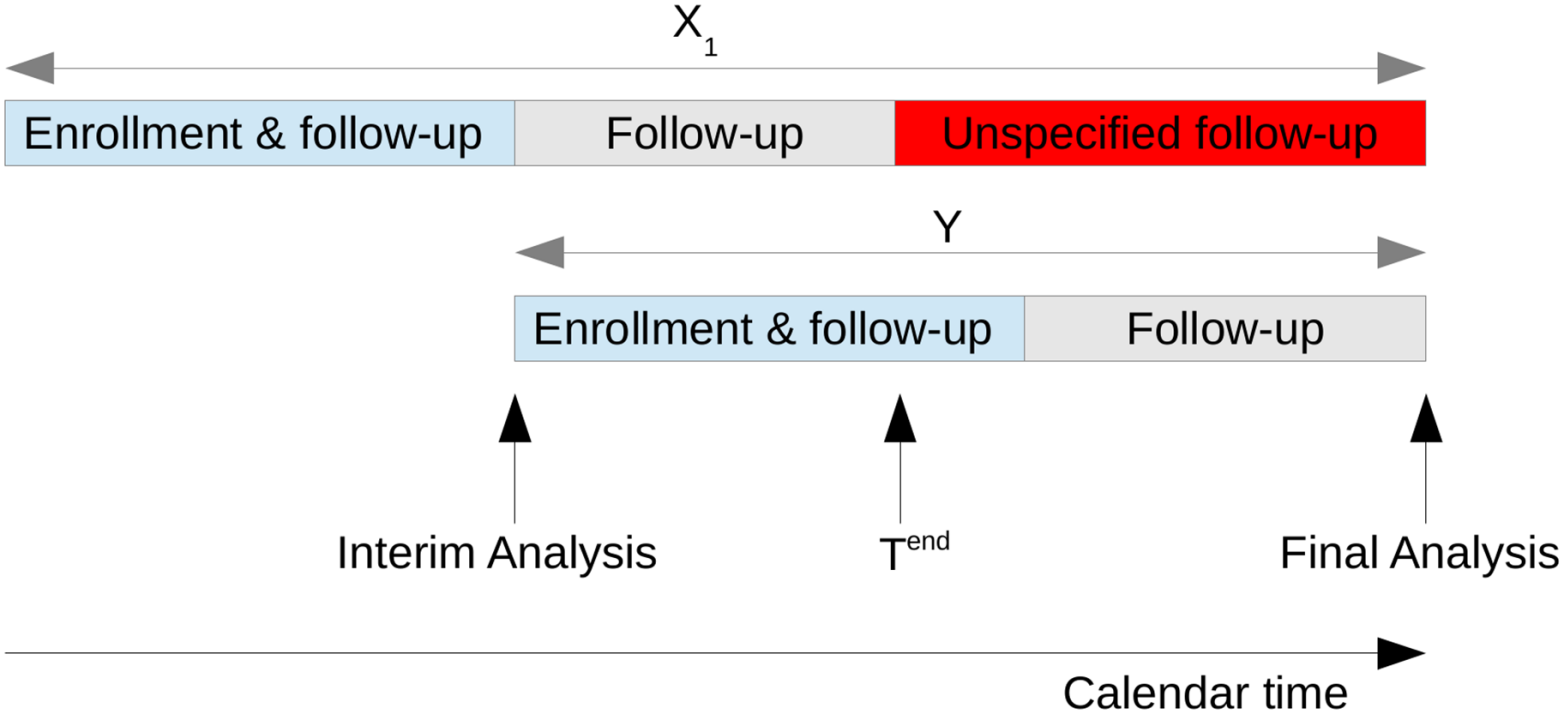
Schematic of a two-stage adaptive trial design with delayed response using patient-wise separation.

The situation is represented pictorially in Fig 2. An unfortunate consequence of pre-fixing *T*^end^ is that this will not, in all likelihood, correspond to the end of follow-up for second-stage patients. All events from first-stage patients that occur after *T*^end^ make no contribution to the statistic Eq (1).

### 2.2 Adaptive Survival Studies

Consider a randomized clinical trial comparing survival times on an experimental treatment, *E*, with those on a control treatment, *C*. For simplicity, we will focus on the logrank statistic for testing the null hypothesis *H*_0_: *θ* = 0 against the one-sided alternative *H*_*a*_: *θ* > 0, where *θ* is the log hazard ratio, assuming proportional hazards. Similar arguments could be applied to the Cox model. Let *D*_1_(*t*) and *S*_1_(*t*) denote the number of uncensored events and the usual logrank score statistic, respectively, based on the data from first-stage patients—those patients recruited prior to the interim analysis—followed up until calendar time *t*, *t* ∈ [0, *T*^max^]. Under the null hypothesis, assuming equal allocation and a large number of events, the variance of *S*_1_(*t*) is approximately equal to *D*_1_(*t*)/4 [25]. The first-stage p-value must be calculated at a prefixed time point *T*^end^:

$$p_{1}=1-\Phi \left[S_{1}\left(T^{\text{end}}\right)/{\left\{D_{1}\left(T^{\text{end}}\right)/4\right\}}^{1/2}\right].$$ (3)

The number of events can be prefixed at *d*_1_, say, with *T*^end^ chosen implicitly

$$T^{\text{end}}≔\min\left\{t:D_{1}\left(t\right)=d_{1}\right\}.$$ (4)

#### Jenkins et al., method

[13] describe a “patient-wise separation” adaptive survival trial, with test statistic Eq (1), first-stage p-value Eq (3) and *T*^end^ defined as in Eq (4). While their focus is on subgroup selection, we will appropriate their method for the simpler situation of a single comparison, where at the interim analysis one has the possibility to adapt the pre-planned number of events from second-stage patients—i.e., those patients recruited post *T*^int^. The weights in Eq (1) are pre-fixed in proportion to the pre-planned number of events to be contributed from each stage, i.e., $w_{1}^{2}=d_{1}/\left(d_{1}+d_{2}\right)$, where *d*_1_ + *d*_2_ is the total originally required number of events. The second-stage p-value corresponds to a logrank test based on second-stage patients, i.e.,

$$p_{2}=1-\Phi \left[S_{2}\left(T_{2}^{*}\right)/{\left\{D_{2}\left(T_{2}^{*}\right)/4\right\}}^{1/2}\right],$$

where $T_{2}^{*}≔\min\{t:D_{2}\left(t\right)=d_{2}^{*}\}$ with *S*_2_(*t*) and *D*_2_(*t*) defined analogously to *S*_1_(*t*) and *D*_1_(*t*), and where $d_{2}^{*}$ is specified at the interim analysis.

#### Irle and Schäfer method

Instead of explicitly combining stage-wise p-values, [12] employ the closely related “conditional error” approach [3, 4, 26].

They begin by prespecifying a level-*α* test with decision function, *φ*, taking values in {0, 1} corresponding to non-rejection and rejection of *H*_0_, respectively. For a survival trial, this entails specifying the sample size, duration of follow-up, test statistic, recruitment rate, etc. Then, at some not necessarily prespecified timepoint, *T*^int^, an interim analysis is performed. The timing of the interim analysis induces a partition of the trial data, (*X*_1_, *X*_2_), where *X*_1_ and *X*_2_ denote the data from patients recruited prior- *T*^int^ and post- *T*^int^, respectively, followed-up until time *T*^max^. For a standard log-rank test, the decision function is

$$\varphi \left(X_{1},X_{2}\right)=1\left[S\left(T^{\text{end}}\right)/{\left\{D(T^{\text{end}})/4\right\}}^{1/2}>\Phi^{-1}\left(1-\alpha \right)\right],$$ (5)

where *D*(*T*^end^) and *S*(*T*^end^) denote the number of uncensored events and the usual logrank score statistic, respectively, based on data from all patients followed-up until time *T*^end^: = min{*t* : *D*(*t*) = *d*} for some prespecified number of events *d*.

At the interim analysis, the general idea is to use the unblinded first-stage data $x_{1}^{\text{int}}$ to define a second-stage design, *δ*, without the need for a prespecified adaptation strategy. Again, the definition of *δ* includes factors such as sample size, follow-up period, recruitment rate, etc., in addition to a second-stage decision function *ψ*: ℝ^*m*^ → {0, 1} based on second-stage data *Y* ∈ ℝ^*m*^. Ideally, one would like to choose *ψ* such that $E_{H_{0}}\left(\psi |X_{1}^{\text{int}}=x_{1}^{\text{int}}\right)=E_{H_{0}}\left(\varphi |X_{1}^{\text{int}}=x_{1}^{\text{int}}\right)$, as this would ensure that

$$E_{H_{0}}(\psi )=E_{H_{0}}\left\{E_{H_{0}}\left(\psi |X_{1}^{\text{int}}\right)\right\}=E_{H_{0}}\left\{E_{H_{0}}\left(\varphi |X_{1}^{\text{int}}\right)\right\}=E_{H_{0}}(\varphi )=\alpha ,$$ (6)

i.e., the overall procedure controls the type I error rate at level *α*. Unfortunately, this approach is not directly applicable in a survival trial where $X_{1}^{\text{int}}$ contains short-term data from first-stage patients surviving beyond *T*^int^. This is because it is impossible to calculate $E_{H_{0}}\left(\varphi |X_{1}^{\text{int}}=x_{1}^{\text{int}}\right)$ and $E_{H_{0}}\left(\psi |X_{1}^{\text{int}}=x_{1}^{\text{int}}\right)$, owing to the unknown joint distribution of survival times and the secondary endpoints already observed at the interim analysis. One may, however, condition on *X*_1_ rather than on $X_{1}^{\text{int}}$ and choose *ψ* such that *E*_*H*_0__(*ψ* ∣ *X*_1_ = *x*_1_) = *E*_*H*_0__(*φ* ∣ *X*_1_ = *x*_1_), thus ensuring type I error control following the same argument as Eq (6). For example, it is possible to extend patient follow-up and use the second-stage decision function

$$\psi \left(X_{2}\right)=1\left[S\left(T^{*}\right)/{\left\{D(T^{*})/4\right\}}^{1/2}\geq b^{*}\right],$$ (7)

where T*: = min{*t* : *D*(*t*) = d*}, d* ≥ *d* is chosen at the interim analysis, and b* is a cutoff value that must be determined. [12] show that, asymptotically,

$$E_{H_{0}}\left\{\varphi |X_{1}=x_{1}\right\}=E_{H_{0}}\left\{\varphi |S_{1}\left(T^{\text{end}}\right)=s_{1}\right\}$$

and

$$E_{H_{0}}\left\{\psi |X_{1}=x_{1}\right\}=E_{H_{0}}\left\{\psi |S_{1}\left(T^{*}\right)=s_{1}^{*}\right\}.$$

In each case, calculation of the right-hand-side is facilitated by the asymptotic result that, assuming equal allocation under the null hypothesis, for *t* ∈ [0, *T*^max^],

$$\left(\begin{array}{c} S_{1}\left(t\right)\\ S\left(t\right)-S_{1}\left(t\right) \end{array}\right)∼N\left(\left(\begin{array}{c} 0\\ 0 \end{array}\right),\left(\begin{array}{cc} D_{1}\left(t\right)/4 & 0\\ 0 & \left\{D\left(t\right)-D_{1}\left(t\right)\right\}/4 \end{array}\right)\right).$$ (8)

One remaining subtlety is that $E_{H_{0}}\{\psi |S_{1}\left(T^{*}\right)=s_{1}^{*}\}$ can only calculated at calendar time T*, where T* > *T*^int^. Determination of b* must therefore be postponed until this later time.

Using result Eq (8), it is straightforward to show that *ψ* = 1 if and only if *Z* > Φ^−1^(1 − *α*), where *Z* is defined as in Eq (1) with *p*_1_ defined as in Eq (3), the second-stage p-value function defined as

$$p_{2}\left(Y\right)=1-\Phi \left(\left\{S\left(T^{*}\right)-S_{1}\left(T^{*}\right)\right\}/\left[\left\{D\left(T^{*}\right)-D_{1}\left(T^{*}\right)\right\}/4\right]{\;}^{1/2}\right),$$ (9)

and the specific choice of weighting $w_{1}^{2}=D_{1}\left(T^{\text{end}}\right)/D\left(T^{\text{end}}\right)$. Full details are provided in supplementary material (S2 File).

**Remark 1.** The Irle and Schäfer method uses the same test statistic as the Jenkins et al. method, with a clever way of implicitly defining the weights and the end of first-stage follow-up, *T*^end^. It has two potential advantages. Firstly, the timing of the interim analysis need not be prespecified—in theory, one is permitted to monitor the accumulating data and at any moment decide that design changes are necessary. Secondly, if no changes to the design are necessary, i.e., the trial completes as planned at calendar time *T*^end^, then the original test Eq (5) is performed. In this special case, no events are ignored in the final test statistic.

**Remark 2.** From first glance at Eq (7), it may appear that the events from first-stage patients, occurring after *T*^end^, always make a contribution to the final test statistic. However, this data is still effectively ignored. We have shown in the online supplement that the procedure is equivalent to a p-value combination approach where *p*_1_ depends only on data available at time *T*^end^. In addition, the distribution of *p*_2_ is asymptotically independent of the data from first-stage patients: note that *S*(T*) − *S*_1_(T*) and *S*_2_(T*) are asymptotically equivalent [12]. The procedure therefore fits our description of a “patient-wise separation” design, and the picture is the same as in Fig 2. The first-stage patients have in effect been censored at *T*^end^, despite having been followed-up for longer. This fact has important implications for the choice of d*. If one chooses d* based on conditional power arguments, one should be aware that the effective sample size has not increased by d* − *d*. Rather, it has increased by d* − *d* − {*D*_1_(T*) − *D*_1_(*T*^end^)}, which could be very much smaller.

**Remark 3.** A potential disadvantage of the Irle and Schäfer method compared to the Jenkins et al. method is that one is not permitted to adapt any aspect of the recruitment process prior to time *T*^end^. Contrary to what is claimed in [12], it is not valid to extend the recruitment period (or speed up recruitment as in the example they give) to reach an increased number of events d* within the originally planned trial duration. This is because *T*^end^ is defined implicitly as *T*^end^: = min{*t* : *D*(*t*) = *d*} under the assumptions of the original design. Therefore *T*^end^ is unobservable if the recruitment process is changed in response to the interim data. [27] discuss this issue further.

### 2.3 Hypothesis tests based on all available follow-up data

Suppose that the trial continues until calendar time T* ∈ (*T*^end^, *T*^max^). Data from first-stage patients—those patients recruited prior to *T*^int^—accumulating between times *T*^end^ and T* should be ignored. In this section we will investigate what happens, in a worst case scenario, if this illegitimate data is naively incorporated into the adaptive test statistic Eq (1). Specifically, we find the maximum type I error associated with the test statistic

$$Z^{*}=w_{1}S_{1}\left(T^{*}\right)/{\left\{D_{1}\left(T^{*}\right)/4\right\}}^{1/2}+w_{2}\Phi^{-1}\left(1-p_{2}\right).$$ (10)

Since T* depends on the interim data in a complicated way, the null distribution of Eq (10) is unknown. One can, however, consider properties of the stochastic process

$$Z\left(t\right)=w_{1}S_{1}\left(t\right)/{\left\{D_{1}\left(t\right)/4\right\}}^{1/2}+w_{2}\Phi^{-1}\left(1-p_{2}\right),\;t\in \left[T^{\text{end}},T^{\max}\right].$$

In other words, we consider continuous monitoring of the logrank statistic based on first-stage patient data. The worst-case scenario assumption is that the responses on short-term secondary endpoints, available at the interim analysis, can be used to predict the exact calendar time the process *Z*(*t*) reaches its maximum. In this case, one could attempt to engineer the second stage design such that T* coincides with this timepoint, and the worst-case type I error rate is therefore

$$P_{H_{0}}\left\{\max_{T^{\text{end}}\leq t\leq T^{\max}}Z\left(t\right)>\Phi^{-1}\left(1-\alpha \right)\right\}.$$ (11)

Although the worst-case scenario assumption is clearly unrealistic, Eq (11) serves as an upper bound on the type I error rate. It can be found approximately via standard Brownian motion results. Let *u*: = *D*_1_(*t*)/*D*_1_(*T*^max^) denote the information time at calendar time *t*, and let *S*_1_(*u*) denote the logrank score statistic based on first-stage patients, followed-up until information time *u*. It can be shown that *B*(*u*): = *S*_1_(*u*)/ {*D*_1_(*T*^max^)/4}^1/2^ behaves asymptotically like a Brownian motion with drift *ξ*: = *θ* {*D*_1_(*T*^max^)/4}^1/2^[28]. We wish to calculate

$$\begin{array}{l} \;P_{\theta =0}\left\{\underset{T^{\text{end}}\leq t\leq T^{\text{max}}}{\text{max}}Z(t)>\Phi^{-1}(1-\alpha )\right\}\\ =\int_{0}^{1}P_{\theta =0}\left[\underset{u_{1}\leq u\leq 1}{\text{max}}B(u)>u^{1/2}w_{1}^{-1}\left\{\Phi^{-1}(1-\alpha )-w_{2}\Phi^{-1}(1-p_{2})\right\}\right]\;\text{d}p_{2}, \end{array}$$ (12)

where *u*_1_ = *D*_1_(*T*^end^)/*D*_1_(*T*^max^). While the integrand on the right-hand-side is difficult to evaluate exactly, it can be found to any required degree of accuracy by replacing the square root stopping boundary with a piecewise linear boundary [29].

The two parameters that govern the size of Eq (11) are *w*_1_ and *u*_1_. Larger values of *w*_1_ reflect an increased weighting of the first-stage data, which increases the potential inflation. In addition, a low value for *u*_1_ increases the window of opportunity for stopping on a random high. Table 1 shows that for a nominal *α* = 0.025 level test, the worst-case type I error can be up to 15% when *u*_1_ = 0.1 and *w*_1_ = 0.9. As *u*_1_ → 0 the worst-case type I error rate tends to 1 for any value of *w*_1_ > 0 [30].

**Table 1:** Worst case type I error for various choices of weights and information fractions. Nominal level *α* = 0.025 one-sided.

	*u*_1_								
*w*_1_	0.1	0.2	0.3	0.4	0.5	0.6	0.7	0.8	0.9
0.1	0.052	0.047	0.044	0.041	0.039	0.037	0.035	0.033	0.030
0.2	0.067	0.059	0.054	0.050	0.046	0.043	0.039	0.036	0.032
0.3	0.081	0.070	0.062	0.057	0.052	0.047	0.043	0.039	0.034
0.4	0.094	0.080	0.071	0.063	0.057	0.052	0.046	0.041	0.036
0.5	0.106	0.089	0.078	0.069	0.062	0.056	0.050	0.044	0.037
0.6	0.119	0.098	0.085	0.075	0.067	0.059	0.053	0.046	0.038
0.7	0.131	0.107	0.092	0.081	0.072	0.063	0.055	0.048	0.040
0.8	0.143	0.116	0.100	0.087	0.076	0.067	0.058	0.050	0.041
0.9	0.155	0.125	0.106	0.092	0.081	0.070	0.061	0.052	0.042

#### A full-data guaranteed level-*α* test

If one is unprepared to give up the guarantee of type I error control, an alternative test can be found by increasing the cut-off value for Z* from Φ^−1^(1 − *α*) to k* such that

$$\int_{0}^{1}P_{\theta =0}\left[\max_{u=u_{1}}^{1}B\left(u\right)>u^{1/2}w_{1}^{-1}\left\{k^{*}-w_{2}\Phi^{-1}\left(1-p_{2}\right)\right\}\right]\;d\;p_{2}=\alpha .$$

## 3 Results

### 3.1 Clinical trial example

The upper bound on the type I error rate varies substantially across *w*_1_ and *u*_1_. To give an indication of what can be expected in practice, consider a simplified version of the trial described in [12]. A randomized trial is set up to compare chemotherapy (C) with a combination of radiotherapy and chemotherapy (E). The anticipated median survival time on C is 14 months. If E were to increase the median survival time to 20 months then this would be considered a clinically relevant improvement. Assuming exponential survival times, this gives anticipated hazard rates *λ*_*C*_ = 0.050 and *λ*_*E*_ = 0.035, and a target log hazard ratio of *θ*_*R*_ = −log(*λ*_*E*_/*λ*_*C*_) ≈ 0.36. If the error rates for testing *H*_0_ : *θ* = 0 against *H*_*a*_ : *θ* = *θ*_*R*_ are *α* = 0.025 (one-sided) and *β* = 0.2, the required number of deaths (assuming equal allocation) is

$$d=4{\left.[\left\{\Phi^{-1}\left(1-\alpha \right)+\Phi^{-1}\left(1-\beta \right)\right\}/\theta_{R}\right]}^{2}\approx 248.$$

The relationship between the required number of events and the sample size depends on the recruitment pattern, and we will consider two scenarios. In our “slow recruitment” scenario, patients are recruited uniformly at a rate of 8 per month for a maximum of 60 months with an interim analysis performed at 23 months. In our“fast recruitment” scenario, patients are recruited uniformly at a rate of 50 per month for a maximum of 18 months with an interim analysis after 8 months. In both cases, the only adaptation we allow at the interim analysis is to increase the number of events. Recruitment must continue as planned but the follow-up period may be extended. The maximum duration of the trial is restricted to 100 months in the first case and 30 months in the second case.

**Fig 3:**
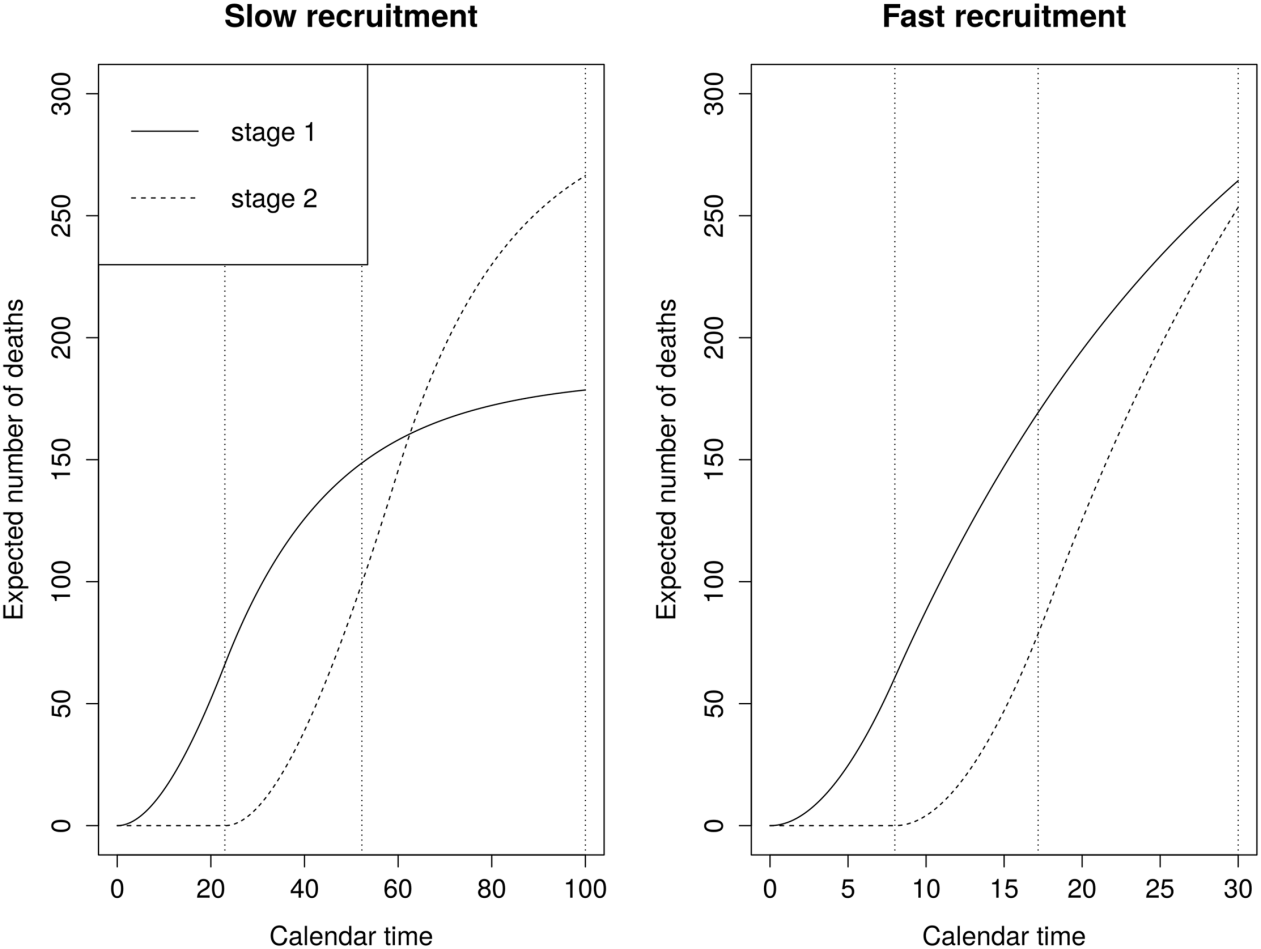
Expected total number of events as a function of time based on exponential survival with hazard rates *λ*_*C*_ = 0.05 and *λ*_*E*_ = 0.035. Slow recruitment: 8 patients per month for a maximum of 60 months. Fast recruitment: 50 patients per month for a maximum of 18 months. Vertical lines are at *T*^int^, *T*^end^ and *T*^max^.

Fig 3 shows the expected number of events as a function of time for both scenarios assuming exponentially distributed survival times with hazards equal to the planned values.

The maximum type I error inflation, determined via *w*_1_ and *u*_1_, will depend on the observed number of events from first- and second-stage patients at calendar times *T*^int^ and *T*^end^. However, the expected pattern of events in Fig 3 provide some indication. In the slow recruitment scenario, we expect to recruit 179 patients by the time of the interim analysis. We also expect 149 of the first 248 events to come from patients recruited prior to the interim analysis. These numbers would give *w*_1_ = (149/248)^1/2^, *u*_1_ = 149/179 and, according to Eq (12), max *α* = 0.044. For the fast recruitment scenarios the respective quantities are *w*_1_ = (169/248)^1/2^, *u*_1_ = 169/264 and max *α* = 0.060.

#### On the efficiency of the full-data level-*α* test

**Fig 4:**
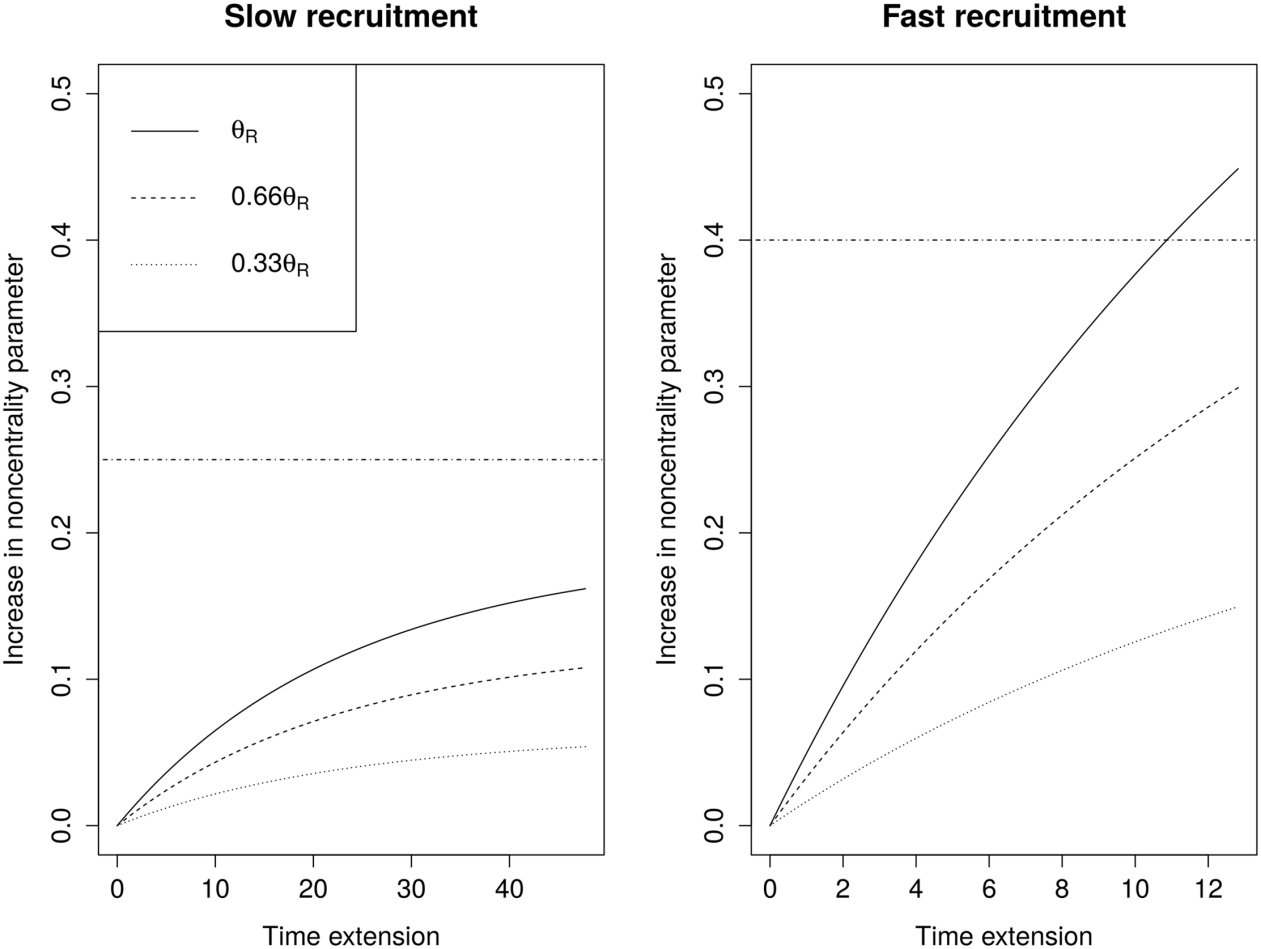
Difference between the noncentrality parameters of the adaptive test statistics *Z*(T*) and *Z*(*T*^end^) as a function of the time extension T* − *T*^end^ ∈ [0, *T*^max^ − *T*^end^]. Horizontal lines are drawn at k* − Φ^−1^(0.975), where k* denotes the cut-off value of the full-data guaranteed level-*α* test, and Φ denotes the standard normal distribution function.

Consider the full-data guaranteed level-*α* test defined above. Recall that this test has the advantage of allowing interim decision making to be based on all available data whilst using a final test statistic that takes account of all observed event times. Unfortunately, this advantage is likely to be outweighed by the loss in power resulting from the increased cut-off value, as can be seen in Fig 4. The difference between the noncentrality parameters of *Z*(T*) and *Z*(*T*^end^) is plotted against the time extension T* − *T*^end^ for various choices of *θ*. In the slow recruitment scenario the increase in the noncentrality parameter is outweighed by the increase in the cut-off value, even when the log-hazard ratio is as large as was expected in the planning phase. In the fast recruitment setting, it is possible for the increase in the noncentrality parameter to exceed the increase in the cut-off value when the trial is extended substantially. However, the trial would typically only need to be increased substantially if the true effect size were lower than planned. And in this case (*θ* ≤ 0.66*θ*_*R*_) one can see that the increased cut-off value still dominates.

## 4 Discussion

Unblinded sample-size recalculation has been criticized for its lack of efficiency relative to classical group sequential designs [31, 32]. If the recalculation is made on the basis of an early estimate of treatment effect, the final sample size is likely to have high variability [33], and, in addition, the test decision is based on a non-sufficient statistic. [34] show how, for a given re-estimation rule, a classical group sequential design can be found with an essentially identical power function but lower expected sample size.

In response to these arguments [35] emphasize that “the real benefit of the adaptive approach arises through the ability to invest sample resources into the trial in stages”. An efficient group sequential trial, on the other hand, requires a large up-front sample size commitment and aggressive early stopping boundaries. From the point of view of the trial sponsor, the added flexibility may in some circumstances outweigh the loss of efficiency.

In this paper we have shown that when the primary endpoint is time-to-event, a fully unblinded sample-size recalculation—i.e., a decision based on all available efficacy and safety data—has additional drawbacks not considered in the aforementioned literature. Recently proposed methods [12, 13] share the common disadvantage that some patients’ event times are ignored in the final test statistic. This is usually deemed unacceptable by regulators. Furthermore, it is the long-term data of patients recruited prior to the interim analysis that is ignored, such that more emphasis is put on early events in the final decision making. This neglect becomes more serious, therefore, if the hazard rates differ substantially only at large survival times. Note, however, that a standard logrank test would already be inefficient in this scenario [36].

The relative benefit of the Irle and Schäfer method [12], in comparison with that of Jenkins et al. [13], is that the timing of the interim analysis need not be pre-specified and, in addition, the method is efficient if no design changes are necessary. On the other hand, the Irle and Schäfer method has the serious practical flaw that it is not permissible to change any aspect of the recruitment process in response to the interim data.

Confirmatory clinical trials with time-to-event endpoints appear to be one of the most important fields of application of adaptive methods [37]. It is therefore especially important that investigators considering an unblinded sample size re-estimation in this context are aware of the additional issues involved. We have shown that all considered procedures will require giving up an important statistical property—a situation summarized succinctly in Table 2.

**Table 2:** Trade-off involved in choosing between methods when extending the follow-up period of a survival trial. Methods considered: (A), data is combined assuming independent stage-wise increments; (B), patient-wise separation with pre-fixed end of first-stage follow-up; (C), naive patient-wise separation without pre-fixed end of first-stage follow-up; and (D), patient-wise separation using the full-data guaranteed level-*α* test.

	Strict type I error control	All data available for interim decisions	All events included in test statistic	Relative power
(A) Ind. Increments	√	×	√	√
(B) *Z*(*T*^end^) > *z*_1−*α*_	√	√	×	√
(C) *Z*(T*) > *z*_1−*α*_	×	√	√	√
(D) *Z*(T*) > k*	√	√	√	×

The relevance of these issues is highlighted by the recently published VALOR trial in acute myeloid leukaemia [38]. Treatment effect estimates from phase II data suggested that 375 events might be sufficient to confirm efficacy. However, there is always uncertainty surrounding such an estimate. A smaller effect size—corresponding to upwards of 500 required events—would still be clinically meaningful, but funding such a trial was beyond the resources of the study sponsor. The solution was to initiate the trial with the smaller sample size but plan an interim analysis, whereby promising results would trigger additional investment. In this case, the interim decision rules were pre-specified and, upon observing a promising hazard ratio after 173 events, the total required number of events was increased to 562. The final analysis was based on a weighted combination of log-rank statistics, corresponding to method (A) in Table 2. It is important to emphasize that the validity of this approach relies on the second-stage sample size being a function of the interim hazard ratio. Had other information—e.g., disease progressions—played a part in the interim decision making, then the type I error rate could have been compromised as described in this paper.

While statistical theory can be developed to control the type I error rate given certain model assumptions, there is always the potential for “operational bias” to enter an adaptive trial. FDA draft guidance [39] emphasizes the need to shield investigators as much as possible from knowledge of the adaptive changes. The very knowledge that sample size has been increased—implying a “promising” interim effect estimate—could lead to changes of behavior in terms of treating, managing, and evaluating study participants. As a minimum, the European Medicines Agency requires that the primary analysis “be stratified according to whether patients were randomized before or after the protocol amendment” [40]. Aside from the regulatory importance, it is also in the sponsor’s interest to minimize operational bias when trial outcomes will influence significant investment decisions [41]. For a further discussion on the regulatory and logistical challenges sponsors may face we refer to [6, 19].

We have focussed our attention on the type I error control and power of the various procedures. Estimation of the treatment effect size following an adaptive survival trial is also an important topic. Current available methods can be found in [8], [42] and [43].

## Supporting Information

S1 File R code to reproduce results. (R)

S2 File The connection between conditional error and the combination test. (PDF)
