# Supplementary material for: Sample Size Reassessment and Hypothesis Testing in Adaptive Survival Trials
Source: PLoS One. 2016 Feb 10;11(2):e0146465. doi: 10.1371/journal.pone.0146465 (PMC4749572; doi:10.1371/journal.pone.0146465)
Supplement: S2 File — (PDF) [file pone.0146465.s002.pdf]

Connection between conditional error and combination test.

The cut-off b^* satisfies

$$
\begin{aligned}
&E_{H_0}\left\{\varphi \mid S_1(T^{\text{end}}) = s_1\right\} \\
&= P_{H_0}\left\{S(T^*)/(d^*/4)^{1/2} \geq b^* \mid S_1(T^*) = s_1^*\right\} \\
&= P_{H_0}\left(\{S(T^*) - S_1(T^*)\} / \left[\{d^* - D_1(T^*)\}/4\right]^{1/2} \geq c^* \mid S_1(T^*) = s_1^*\right],
\end{aligned}
$$

which implies that $c^* = \Phi^{-1}\left[1 - E_{H_0}\{\varphi \mid S_1(T) = s_1\}\right]$. Therefore,

$$
\begin{aligned}
\psi = 1 &\Leftrightarrow S(T^*)/(d^*/4)^{1/2} \geq b^* \\
&\Leftrightarrow \{S(T^*) - S_1(T^*)\} / \left[\{d^* - D_1(T^*)\}/4\right]^{1/2} \geq c^* \\
&\Leftrightarrow \Phi^{-1}(1-p_2) \geq \Phi^{-1}\left[1 - E_{H_0}\left\{\varphi \mid S_1(T^{\text{end}}) = s_1\right\}\right] \\
&\Leftrightarrow p_2 \leq E_{H_0}\left\{\varphi \mid S_1(T^{\text{end}}) = s_1\right\}.
\end{aligned}
$$

The conditional error probability, $E_{H_0}\left\{\varphi \mid S_1(T^{\text{end}}) = s_1\right\}$, can be found from the joint distribution of $S_1(T^{\text{end}})$ and $S(T^{\text{end}})$. Omitting the argument T^{end} from S_1, S, D_1 and D:

$$
\begin{aligned}
E_{H_0}\{\varphi \mid S_1 = s_1\} &= P_{H_0}\left\{S/(D/4)^{1/2} > \Phi^{-1}(1-\alpha) \mid S_1 = s_1\right\} \\
&= P_{H_0}\Big[2(S - S_1)/(D - D_1)^{1/2} > \Phi^{-1}(1-\alpha)\{D/(D-D_1)\}^{1/2} \\
&\qquad\qquad -2S_1/(D-D_1)^{1/2} \mid S_1 = s_1\Big] \\
&= 1 - \Phi\left[\Phi^{-1}(1-\alpha)\{D/(D-D_1)\}^{1/2} - \Phi^{-1}(1-p_1)\{D_1/(D-D_1)\}^{1/2}\right]
\end{aligned}
$$

and therefore $p_2 \leq E_{H_0}\{\varphi \mid S_1(T) = s_1\}$ if and only if

$$
\left\{D_1(T^{\text{end}})/d\right\}^{1/2}\Phi^{-1}(1-p_1) + \left[\left\{d - D_1(T^{\text{end}})\right\}/d\right]^{1/2}\Phi^{-1}(1-p_2) \geq \Phi^{-1}(1-\alpha).
$$
